# Prediction and analysis of periprocedural complications associated with endovascular treatment for unruptured intracranial aneurysms using machine learning

**DOI:** 10.3389/fneur.2022.1027557

**Published:** 2022-10-12

**Authors:** Zhongbin Tian, Wenqiang Li, Xin Feng, Kaijian Sun, Chuanzhi Duan

**Affiliations:** ^1^National Key Clinical Specialty, Engineering Technology Research Center of Education Ministry of China, Guangdong Provincial Key Laboratory on Brain Function Repair and Regeneration, Neurosurgery Institute, Department of Neurosurgery, Zhujiang Hospital, Southern Medical University, Guangzhou, China; ^2^Department of Neurosurgery, The First Affiliated Hospital of Zhengzhou University, Zhengzhou, China

**Keywords:** intracranial aneurysm, endovascular treatment, periprocedural complication, machine learning (ML), feature importance analysis

## Abstract

**Background:**

The management of unruptured intracranial aneurysm (UIA) remains controversial. Recently, machine learning has been widely applied in the field of medicine. This study developed predictive models using machine learning to investigate periprocedural complications associated with endovascular procedures for UIA.

**Methods:**

We enrolled patients with solitary UIA who underwent endovascular procedures. Periprocedural complications were defined as neurological adverse events resulting from endovascular procedures. We incorporated three machine learning algorithms into our prediction models: artificial neural networks (ANN), random forest (RF), and logistic regression (LR). The Shapley Additive Explanations (SHAP) approach and feature importance analysis were used to identify and prioritize significant features associated with periprocedural complications.

**Results:**

In total, 443 patients were included. Forty-eight (10.83%) procedure-related complications occurred. In the testing set, the ANN model produced the largest value (0.761) for area under the curve (AUC). The RF model also achieved an acceptable AUC value of 0.735, while the AUC value of the LR model was 0.668. SHAP and feature importance analysis identified distal aneurysm, aneurysm size and treatment modality as most significant features for the prediction of periprocedural complications following endovascular treatment for UIA.

**Conclusion:**

Periprocedural complications after endovascular treatment for UIA are not negligible. Prediction of periprocedural complications *via* machine learning is feasible and effective. Machine learning can serve as a promising tool in the decision-making process for UIA treatment.

## Introduction

The prevalence of unruptured intracranial aneurysm (UIA) in the adult population is about 3–7% (1, 2). The rupturing of UIAs usually results in subarachnoid hemorrhage, which is associated with a high rate of mortality and morbidity (3). In recent decades, endovascular treatment has become the first-line of treatment for intracranial aneurysm and has achieved satisfactory outcomes (4). However, most UIAs have a low annual risk of rupture, and complications related to the endovascular treatment of UIAs should not be neglected (5, 6). It remains controversial whether UIA should be treated or not. For these reasons, the risk of complications from endovascular treatment should be carefully weighed against the risk of UIA rupture. Establishing a method to identify factors associated with procedure-related complications, and to predict risk from such complications, could provide critical reference guidelines to physicians.

Recent studies have applied machine learning (ML) to the prediction of intracranial aneurysm rupture and outcome after endovascular treatment (7–9). When challenged with complex non-linear relationships across large datasets, ML can generate automated decisions that often outperform conventional statistical methods. Liu et al. and Zhu et al. reported promising results from the application of ML techniques to the prediction of aneurysm stability (10, 11). Paliwal et al. and Guédon et al. developed ML models to predict occlusion outcomes from aneurysms following flow diverter deployment (12, 13). However, research on the prediction of periprocedural complications from endovascular treatment is still scarce.

In this study, we exploited three ML algorithms to develop predictive models for periprocedural complications after endovascular treatment: artificial neural networks (ANN), random forest (RF), and logistic regression (LR). We then compared their prediction performance. To improve model interpretability and identify significant factors associated with periprocedural complications, we applied the Shapley Additive Explanations (SHAP) method and feature importance analysis. Our results provide physicians with reference guidelines for the management of UIAs.

## Methods

### Patient selection

This retrospective study was approved by the relevant institutional ethics committee, and written informed consent was obtained from patients or their relatives during hospitalization. We included patients with solitary unruptured saccular intracranial aneurysm that were treated endovascularly between January 2016 and December 2019. We adopted the following exclusion criteria: dissecting aneurysm, previous treatment, covered stent deployment, treatment performed by parent artery occlusion, and the existence of a brain arteriovenous malformation. On the basis of these criteria, we retained 443 cases for this study.

### Endovascular procedures

The specific strategy for endovascular treatment was determined by a neurovascular team and was individually tailored to each case. Following general anesthesia, the endovascular procedure was performed. All patients received systemic intravenous heparin. If the team determined that it was necessary to deploy a conventional stent or a flow diverter, the endovascular procedure was preceded by a 5-day dual antiplatelet therapy (100 mg/d of aspirin and 75 mg/d of clopidogrel). If the team opted for a conventional stent, patients were advised to take clopidogrel (75 mg/d) for 6 weeks and aspirin (100 mg/d) for 6 months. If the flow diverter was deployed, the patient would take clopidogrel (75 mg/d) for 3 months and aspirin (100 mg/d) on a permanent basis thereafter.

### Outcome measures

We recorded periprocedural complications that occurred within 30 days of the endovascular procedure. We divided the 443 cases into two groups: complication group and control group. Patients with periprocedural complications were assigned to the complication group. Periprocedural complications were defined as any neurological adverse event (increase in modified Rankin Scale score) resulting from the endovascular treatment. An adverse event was defined as major if the associated neurological deficit lasted longer than 7 days, otherwise it was defined as minor (14).

### Clinical and morphological features

We analyzed the following factors: age, elderly status (>65 years of age), gender, potential risk factors (history of cigarette smoking and alcohol intake, hypertension, cardiovascular disease, hyperlipidemia, diabetes, and previous cerebral ischemic comorbidities), treatment modality (coiling only, stent-assisted coiling, or flow diverter treatment), aneurysm size (maximum size), presence of large aneurysm (size ≥ 10 mm), aneurysm neck size, presence of wide-neck aneurysms (≥4 mm or dome-neck ratio ≤ 2), location (anterior/posterior circulation), shape (defined as irregular if presenting blebs, nipples, or multiple lobes), and presence of distal aneurysm (distally to the Circle of Willis).

### Machine learning model development

We randomly divided data samples into training set (310 cases) and testing set (133 cases) with a 7:3 ratio. Because the dataset was imbalanced between complication and control groups, we applied an adaptive synthetic (ADASYN) sampling method to generate more synthetic data for the minority class (complication group) in the training set (15). After application of ADASYN, the training set was expanded to 553 cases (280 complication cases). We then trained three ML algorithms (ANN, RF, and LR) on the training set with ten-fold cross validation and grid search to optimize hyperparameters for each model. Details of the ML models are provided in Supplementary material. The testing set was used to estimate model performance. Model performance was evaluated *via* receiver operating characteristic (ROC) analysis. To improve model interpretability and investigate important features associated with perioperative complications, we used the SHAP method and feature importance analysis (16). We used the SHAP method to explore important features in ANN models. We used feature score/coefficient to evaluate feature importance in RF and LR models.

### Statistical analysis

We performed statistical analyses using version 22.0 of SPSS (IBM Corp., Armonk, NY, USA). Data are presented as mean and standard deviation for quantitative variables, and as frequency for qualitative variables. We used univariate logistic analysis to analyze risk factors related to periprocedural complications after endovascular procedure for UIA. Statistical significance was defined as p < 0.05.

## Results

### Patient and aneurysm characteristics

We enrolled a total of 443 patients (281 females and 162 males) for this study. Mean age was 55.97 ± 11.41 years. Mean aneurysm size was 6.92 ± 5.08 mm. Of the 443 cases, 75 cases were treated with coil embolization only, 270 with stent-assisted coiling, and 98 with flow diverter therapy.

### Periprocedural complications

In total, 48 (10.83%) procedure-related complications occurred: 4 intraprocedural aneurysm ruptures (0.90%), 2 postprocedural aneurysm ruptures (0.45%), 2 cases of cranial nerve palsy (0.45%), and 40 ischemic events (9.03%). The 40 ischemic events included 26 ischemic strokes, 7 transient ischemic attacks, 4 intra-stent thrombosis and 3 thrombosis resulting from coil migration. Of these 48 procedure-related complications, 27 cases (6.09%) were associated with minor adverse events that resolved on discharge, and 21 (4.74%) were associated with major adverse events.

### Risk factors for periprocedural complications

Table 1 shows results from univariate logistic regression analysis of risk factors for periprocedural complications. The age of the complication group was significantly older than that of the control group (59 ± 12 vs. 56 ± 11, *p* = 0.043), and the complication group included a higher proportion of elderly patients than the control group (35.4% vs. 16.2%, *p* = 0.002). Patients with hypertension and distal aneurysm showed a tendency toward more periprocedural complications (*p* = 0.006 and *p* = 0.045, respectively). Aneurysm size was significantly larger in the complication group than in the control group (8.67 ± 5.54 mm vs. 6.71 ± 4.98 mm, *p* = 0.014), and the complication group had larger aneurysms than the control group (31.3% vs. 16.7%, *p* = 0.016). Moreover, the incidence of periprocedural complications was higher in cases treated by flow diverter therapy (13.3%) or stent-assisted coiling (11.1%) than cases treated by coiling only (6.7%), although this difference did not reach statistical significance (*p* = 0.167 and *p* = 0.265, respectively).

**Table 1:** Results from univariate logistic regression analysis for all variables.

**Characteristics**	**Control group (*N* = 395)**	**Complication group (*N* = 48)**	***P-*value**
Age (y)	55.59 ± 11.25	59.13 ± 12.28	0.043
Elderly	64 (16.2)	17 (35.4)	0.002
Gender (%)			0.276
Male	141 (35.7)	21 (43.8)	
Female	254 (64.3)	27 (56.3)	
Cigarette smoking (%)	41 (10.4)	6 (12.5)	0.653
Alcohol intake (%)	36 (9.1)	7 (14.6)	0.232
Hypertension (%)	195 (49.4)	34 (70.8)	0.006
Hyperlipidemia	108 (27.3)	13 (27.1)	0.970
Cardiovascular disease	17 (4.3)	2 (4.2)	0.965
Diabetes	35 (8.9)	2 (4.2)	0.279
Previous ischemic stroke	106 (26.8)	17 (35.4)	0.212
Potential risk factors (≥2)	165 (41.8)	27 (56.3)	0.058
Aneurysm size (mm)	6.71 ± 4.98	8.67 ± 5.54	0.014
Large aneurysm (%)	66 (16.7)	15 (31.3)	0.016
Neck size (mm)	5.21 ± 3.82	5.80 ± 2.61	0.301
Wide-neck aneurysm	349 (88.4)	47 (97.6)	0.075
Shape (%)			0.731
Regular	305 (77.2)	36 (75.0)	
Irregular	90 (22.8)	12 (25.0)	
Location (%)			0.906
Anterior circulation	364 (92.2)	44 (91.7)	
Posterior circulation	31 (7.8)	4 (8.3)	
Distal aneurysm	109 (27.6)	20 (41.7)	0.045
Treatment modality (%)			
Coiling	70 (17.7)	5 (10.4)	Ref
Stent-assisted coiling	240 (60.8)	30 (62.5)	0.265
Flow diverter	85 (21.5)	13 (27.1)	0.167

### Model performance and identification of important features

**Figure 1:**
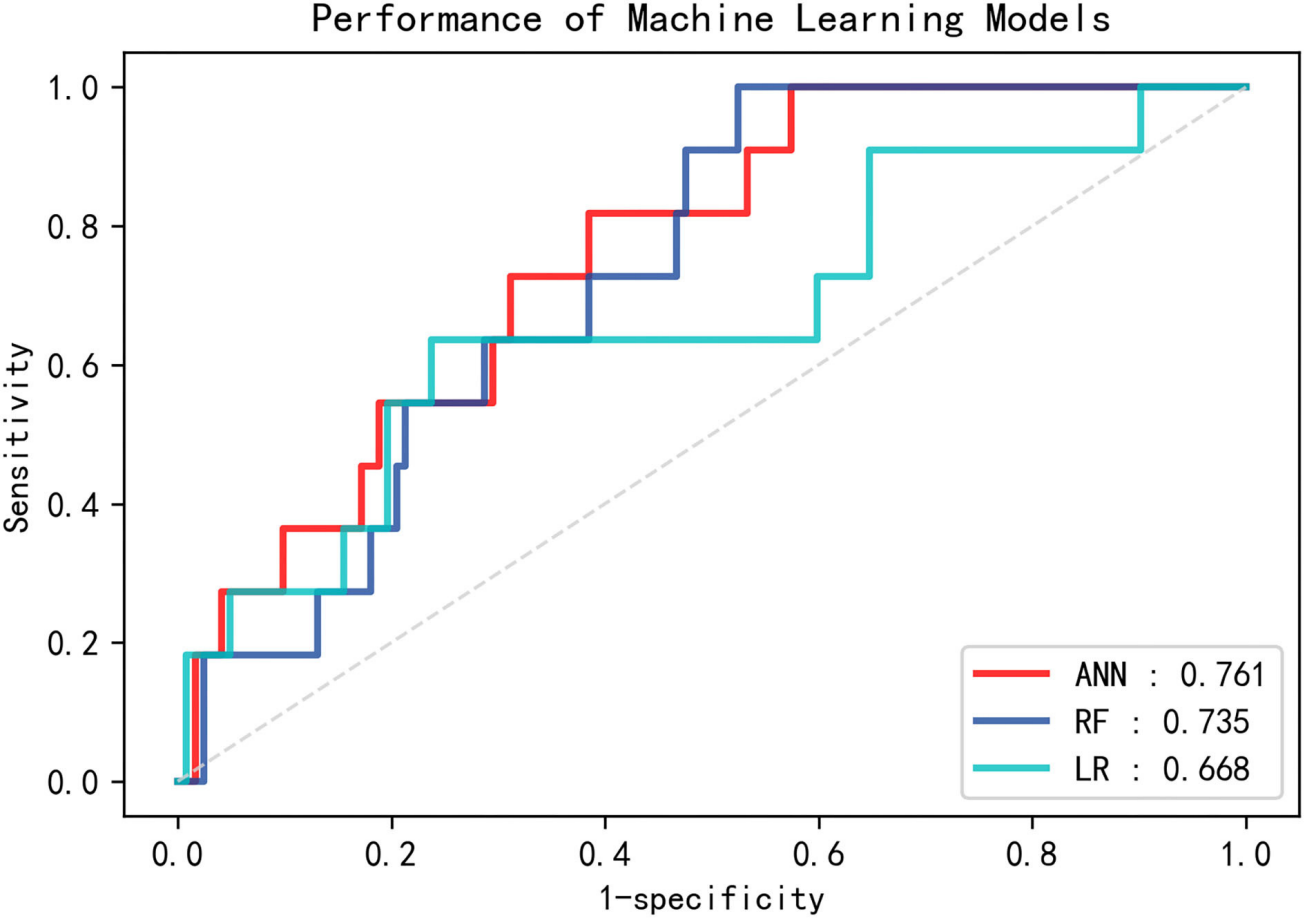
Receiver operating characteristic (ROC) curves for the three machine learning models (ANN, RF, and LR) on the testing set. ANN, artificial neural network; RF, random forest; LR, logistic regression.

With relation to the training set, the area under the curve (AUC) value for the ANN model [0.993; 95% confidence interval (CI) 0.985–0.999] was similar to the AUC value for the RF model (0.999; 95% CI 0.998–1.000), followed by that associated with the LR model (0.768; 95% CI 0.729–0.808). When applied to the testing set, the ANN model produced the highest AUC value (0.761; 95% CI 0.634–0.888; Figure 1). The RF model also achieved an acceptable AUC value (0.735; 95% CI 0.616–0.854), while the AUC for the LR model was 0.668 (95% CI 0.480–0.857).

**Figure 2:**
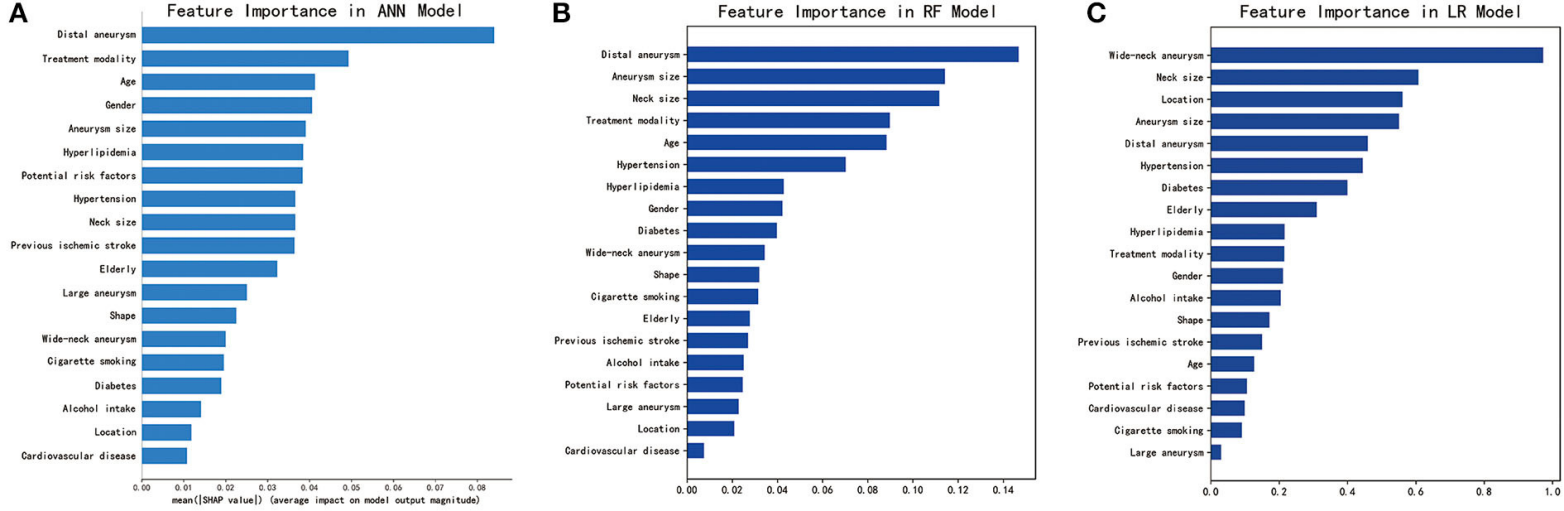
Identification of important features for the three machine learning models. **(A)** SHAP analysis for the ANN model. **(B)** Feature importance analysis for the RF model. **(C)** Feature importance analysis for the LR model. ANN, artificial neural network; RF, random forest; LR, logistic regression. For categorical variables, gender (male), potential risk factors (≥2), location (posterior circulation), and shape (irregular) were analyzed as potential risk factors. Receiver operating characteristic (ROC) curves for the three machine learning models (ANN, RF, and LR) on the testing set. ANN, artificial neural network; RF, random forest; LR, logistic regression.

As shown in Figure 2, SHAP analysis on the ANN model showed that presence of a distal aneurysm and treatment modality were the most important features associated with periprocedural complications. These were also identified as important features for the RF model by feature importance analysis. Aneurysm size was one of the top features for all three ML models. Overall, we identified distal aneurysm, aneurysm size, and treatment modality as important features associated with endovascular treatment.

## Discussion

Periprocedural complications associated with endovascular treatment for UIA represent a source of serious concern for practitioners. In the current study, we developed three ML models to predict these events and investigate risk factors associated with periprocedural complications. First and foremost, our results demonstrate that it is feasible to predict periprocedural complications associated with endovascular treatment using ML. Distal aneurysm, aneurysm size, and treatment modality may be key risk factors associated with endovascular treatment. Our findings may serve as a reference for physicians, and aid their decision-making process prior to UIA treatment.

ML is advantageous in exploring complex non-linear relationships across large datasets, and is a promising tool for clinical decision-making (17). Although many studies have reported successful ML prediction of risk for aneurysm rupture, there is little research on the application of ML to the prediction of periprocedural complications associated with endovascular treatment (18, 19). Ji et al. developed a scoring system for predicting the risk of neurological complications after endovascular treatment of UIAs, but their system was based on only three key factors (aneurysm size, aneurysm location, and cerebral ischemic comorbidity). Their approach may therefore be unsuitable for real-world applications (20). Staartjes et al. explored the feasibility of predicting neurological deficits after microsurgery for UIAs *via* application of ML techniques, and found that these methods support adequate prediction of early clinical endpoints after microsurgery for UIAs (21). However, their study did not include endovascularly treated UIA patients, and their models may therefore perform poorly when applied to such cases. In this study, we developed three ML models to predict perioperative complications associated with endovascular treatment for UIAs. Our results show that the ANN and RF models deliver satisfactory performance, indicating that ML is a valuable tool for prediction of perioperative complications after endovascular treatment for UIAs.

Distal aneurysm is an important predictor of periprocedural complications. In distal aneurysms, diameter of the parent artery and aneurysm size are often relatively small (22). In addition, the parent artery often presents several anatomical variants with numerous perforators or important small vessels that cannot be displayed on digital subtraction angiography. At the same time, sacrificing such vessels can result in neurological deficits (20), and distal location can increase arterial tortuosity. These factors pose serious challenges for successful endovascular treatment. Furthermore, they restrict the movement of endovascular devices, thus resulting in a higher rate of periprocedural complications. In this study, distal aneurysm was the most important feature for both ANN and RF models.

The modality of endovascular treatment has been demonstrated to be closely associated with periprocedural complications. Piotin et al. reported results from 1137 patients treated by coiling only or stent-assisted coiling (23). These authors found that stent-assisted coiling caused more permanent neurologic complications than coiling only (7.4 vs. 3.8%, *p* = 0.64) and a higher procedure-related mortality (4.6 vs. 1.2%, *p* = 0.006). Algra et al. reported that stents are associated with a higher complication risk than coiling (24). Naggara et al. found that the use of a flow-diverter device doubled the risk of unfavorable outcomes compared with simple coil placement (25). In accordance with these previous studies, we found that coiling was the safest treatment modality. Compared with coiling only, both flow-diverter devices and stent-assisted coiling resulted in more periprocedural complications. We also found that treatment modality was one of the most important features for both ANN and RF models, although this result did not reach statistical significance after univariate logistic regression analysis. This apparent discrepancy may be due to the advantage of ML over conventional statistical methods in dealing with complex non-linear relationships across large datasets.

Larger aneurysm size has been reported to be associated with increased risk of periprocedural complications after endovascular treatment (26). Larger aneurysm size increases the complexity of endovascular procedures, and impedes good wall apposition for stent deployment (27). Furthermore, the embolization rate of intracranial aneurysm decreases with increasing aneurysm size, which means that larger aneurysms are more likely to carry residual flow within the coil mass (28–30). Our results confirm and extend these findings by demonstrating that aneurysm size is larger in the complication group compared with the control group. Furthermore, we found that aneurysm size was an important feature for all three ML models.

## Limitations

Our study presents several limitations. Our dataset is relatively small and may involve patient selection bias. Therefore, our results may not generalize well to other patients and settings. Moreover, the synthetic data generated by the ADASYN procedure may not adequately represent less frequent cases. Future verification of our findings and validation of our models will require larger datasets from multiple centers.

## Conclusion

Periprocedural complications after endovascular treatment for UIA can carry substantial consequences for patients. We show that these complications can be successfully predicted using ML models. These models represent promising tools for aiding decision-making prior to UIA treatment.

## Data availability statement

The raw data supporting the conclusions of this article will be made available by the authors, without undue reservation.

## Ethics statement

The studies involving human participants were reviewed and approved by the Ethics Committee of Zhujiang Hospital of Southern Medical University. Written informed consent to participate in this study was provided by the participants' legal guardian/next of kin.

## Author contributions

ZT performed the manuscript writing and statistical analysis. WL processed the data. XF and KS acquired the data. CD conceived and designed the research. All authors contributed to the article and approved the submitted version.

## Funding

This work was funded by the National Key Research Development Program (Grant numbers: 2016YFC1300804 and 2016YFC1300800), the National Natural Science Foundation of China (81974178, 81974177, and 82001300), the Science and Technology Project Foundation of Guangdong province (Grant number: 2016A020215098), and the Key Project of Clinical Research of Southern Medical University (Grant number: LC2016ZD024).

## Conflict of interest

The authors declare that the research was conducted in the absence of any commercial or financial relationships that could be construed as a potential conflict of interest.

## Publisher's note

All claims expressed in this article are solely those of the authors and do not necessarily represent those of their affiliated organizations, or those of the publisher, the editors and the reviewers. Any product that may be evaluated in this article, or claim that may be made by its manufacturer, is not guaranteed or endorsed by the publisher.
