# Supplementary material for: Prediction and analysis of periprocedural complications associated with endovascular treatment for unruptured intracranial aneurysms using machine learning
Source: Front Neurol. 2022 Oct 12;13:1027557. doi: 10.3389/fneur.2022.1027557 (PMC9596813; doi:10.3389/fneur.2022.1027557)
Supplement: Supplementary file 1 [file Data_Sheet_1.docx]

**Machine Learning Models**

ANN is a simple multilayer feed forward neural network based on the back propagation algorithm, which consists of input layer, 5 hidden layers and output layer. Tanh, Relu or Sigmoid were used as the activation function. Meanwhile, dropout function was applied to prevent the model to overfit the training data.

For the RF model, the four optimal hyperparameter values are as follows: n_estimators=24, min_samples_split=2, min_samples_leaf=1, max_depth=10.

The best hyperparameter of LR model were as follows: penalty = ‘l2’ and C = 1.
